# Superior Silencing by 2′,4′-BNA^NC^-Based Short Antisense Oligonucleotides Compared to 2′,4′-BNA/LNA-Based Apolipoprotein B Antisense Inhibitors

**DOI:** 10.1155/2012/707323

**Published:** 2012-09-26

**Authors:** Tsuyoshi Yamamoto, Hidenori Yasuhara, Fumito Wada, Mariko Harada-Shiba, Takeshi Imanishi, Satoshi Obika

**Affiliations:** ^1^Applied Biopharmaceutical Sciences, Graduate School of Pharmaceutical Sciences, Osaka University, 1-6 Yamadaoka, Suita, Osaka 565-0871, Japan; ^2^Department of Molecular Innovation in Lipidology, National Cerebral and Cardiovascular Center Research Institute, 5-7-1 Fujishirodai, Suita, Osaka 565-8565, Japan; ^3^BNA Inc, 7-7-20 Saito-Asagi, Ibaraki, Osaka 567-0085, Japan

## Abstract

The duplex stability with target mRNA and the gene silencing potential of a novel bridged nucleic acid analogue are described. The analogue, 2′,4′-BNA^NC^ antisense oligonucleotides (AONs) ranging from 10- to 20-nt-long, targeted apolipoprotein B. 2′,4′-BNA^NC^ was directly compared to its conventional bridged (or locked) nucleic acid (2′,4′-BNA/LNA)-based counterparts. Melting temperatures of duplexes formed between 2′,4′-BNA^NC^-based antisense oligonucleotides and the target mRNA surpassed those of 2′,4′-BNA/LNA-based counterparts at all lengths. An *in vitro* transfection study revealed that when compared to the identical length 2′,4′-BNA/LNA-based counterpart, the corresponding 2′,4′-BNA^NC^-based antisense oligonucleotide showed significantly stronger inhibitory activity. This inhibitory activity was more pronounced in shorter (13-, 14-, and 16-mer) oligonucleotides. On the other hand, the 2′,4′-BNA^NC^-based 20-mer AON exhibited the highest affinity but the worst IC_50_ value, indicating that very high affinity may undermine antisense potency. These results suggest that the potency of AONs requires a balance between reward term and penalty term. Balance of these two parameters would depend on affinity, length, and the specific chemistry of the AON, and fine-tuning of this balance could lead to improved potency. We demonstrate that 2′,4′-BNA^NC^ may be a better alternative to conventional 2′,4′-BNA/LNA, even for “short” antisense oligonucleotides, which are attractive in terms of drug-likeness and cost-effective bulk production.

## 1. Introduction

Recently designed and synthesized high-performance modified-nucleic-acids (HiPerNAs) such as 2′-*O*-methyl RNA (2′-OMe), 2′-*O*-methoxyethyl RNA (MOE), and 2′,4′-bridged nucleic acid/locked nucleic acid (2′,4′-BNA/LNA) have improved performance compared to phosphorothioate antisense oligonucleotides (AONs). HiPerNAs overcome the systemic antisense effects of these earlier antisense oligonucleotides and show promise as antisense therapeutics for the treatment of a variety of diseases [1–5]. However, more potent and less toxic AONs are required, since several clinical trials of AON drugs carrying HiPerNAs have been recently terminated due to the lack of efficacy or because of safety concerns. In addition, toxicity and delivery problems remain [6–8].

**Figure 1:**
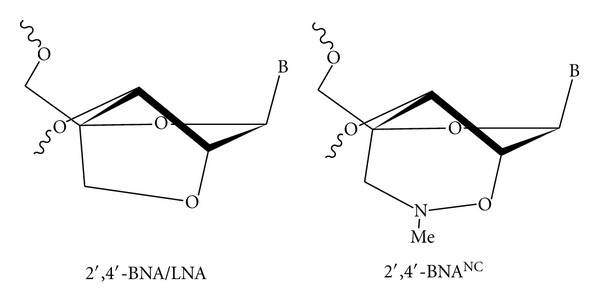
Structures of the BNAs. The chemical structure on the left is the original BNA, 2′,4′-BNA/LNA, and the structure on the right is 2′,4′-BNA^NC^.

We previously described a unique modified nucleic acid, 2′,4′-bridged nucleic acid (2′,4′-BNA; also known as LNA) [9, 10]. Its high therapeutic efficacy is based on the extraordinarily high target binding of the original 2′,4′-BNA/LNA-based AON. 2′,4′-BNA/LNA-based AON is widely accepted as one of the most promising antisense drugs, so fine-tuning the structure of BNA is the key for further improving the therapeutic potency and toxicological properties of AON. In this context, Seth and coworkers developed BNA analogues and elucidated their potency and safety *in vivo*. They reported BNA or LNA analogues with 2′,4′-BNA/LNA-like binding affinities and biological activities with increased nuclease resistance and reduced toxicity [11–14]. We have also reported the synthesis and physicochemical properties of several novel BNAs, including 2′,4′-BNA^COC^ and 2,′4′-BNA^NC^ [15–17], and recently demonstrated the biological activity of a 2′,4′-BNA^NC^ -based AON. Our results indicate that 2′,4′-BNA^NC^ may be a better candidate as an antisense therapeutic [7]. 2′,4′-BNA^NC^ is a six-membered bridged structure containing a hydrophilic aminooxy moiety. A wide range of functional groups can be easily introduced, and the introduction of nitrogen atoms would improve the stability of the duplex by reducing repulsions between phosphates in the backbone [18, 19] (Figure 1). 2′,4′-BNA^NC^-modified oligonucleotides have very high RNA affinity, similar to or even higher than their 2′,4′-BNA/LNA counterparts. Moreover, 2′,4′-BNA^NC^-modified oligonucleotides are more resistant to endonucleolytic cleavage by nucleases than their 2′,4′-BNA/LNA counterparts [17]. Although there is limited information regarding the biological activity or therapeutic potency of 2′,4′-BNA^NC^-based AONs, we have demonstrated the high systemic effects and safety of 2′,4′-BNA/LNA- and 2′,4′-BNA^NC^-based 20-nucleotide-long (20-nt-long) AONs that target PCSK9 mRNA [7]. Additionally, Prakashet al. independently showed the high potency and the nontoxicity of 2′,4′-BNA^NC^-based AONs [11].

 Straarup et al. recently shortened the length of 2′,4′-BNA/LNA-based phosphorothioate AONs to eliminate the latent potency of 2′,4′-BNA/LNA drugs [20]. These short phosphorothioate AONs contain central 6- to 10-nt-long DNA regions flanked by terminal 2- to 4-nt-long 2′,4′-BNA/LNA segments. These short (12- to 14-nt) AONs would be beneficial in terms of target specificity. The introduction of only small numbers of modifications into short 2′,4′-BNA/LNA-based AONs can greatly increase target affinity. Thus, short 2′,4′-BNA/LNA phosphorothioate AONs can minimize length-dependent disadvantages such as phosphorothioate-related protein binding and RNase H inactivation [21–23] while maintaining satisfactory affinity and specificity. Additionally, short AONs are easier to produce on a bulk scale and could exhibit more drug-like characteristics. Based on the assumption that the strand-shortening strategy is also applicable to 2′,4′-BNA^NC^-based AONs, we shortened 2′,4′-BNA^NC^-based phosphorothioate AONs and directly compared their silencing activities against the corresponding 2′,4′-BNA/LNA-based apolipoprotein B (apoB)-targeting AONs.

## 2. Materials and Methods

### 2.1. Oligonucleotides

A series of 2′,4′-BNA/LNA-based antisense 10- to 20-nucleotide-long phosphorothioate gapmers reported previously by Straarup et al. [20] were prepared and used in this study. These AONs were designed with complementary target sites for both cynomolgus monkey and human apoB mRNA sequences. The 10- to 16-nt-long AONs can also target murine apoB mRNA (GenBank accession number NM_000384 and NM_009693 for human and mouse apoB mRNA, resp.). Additionally, we prepared 2′,4′-BNA^NC^-based counterparts in which all the 2′,4′-BNA/LNAs were substituted by 2′,4′-BNA^NC^. The synthesis of 2′,4′-BNA^NC^ with pyrimidine bases was previously reported [16, 17]; the synthesis of 2′,4′-BNA^NC^ with purine bases is currently being optimized and will be reported elsewhere. All the modified oligonucleotides were synthesized by Gene Design, Inc. (Ibaraki, Osaka, Japan) using standard phosphoramidite procedures and purified using HPLC.

### 2.2. Thermal Melting Study of Duplexes

UV melting experiments were carried out using a SHIMAZU UV-1650 spectrometer equipped with a *T*_*m*_ analysis accessory. Equimolar amounts of two single-stranded oligonucleotides were dissolved in 10 mM sodium phosphate buffer (pH 7.2) containing 100 mM NaCl to give a final strand concentration of 4.0 *μ*M. The mixture was annealed by heating at 90°C followed by slow cooling to room temperature. The melting profile was recorded at 260 nm in the forward and reverse direction from 5 to 90°C at a scan rate of 0.5°C/min.

### 2.3. *In Vitro* Transfection Procedures

For AON transfection experiments, Huh-7 cells were seeded at 15 × 10^4^ cells per well in 12-well plates. AONs were transfected by using Lipofectamine 2000 (Invitrogen, Carlsbad, CA) according to the manufacturer's procedures. After a 4-hour transfection, cells were washed with PBS, fresh medium was added, and the cells were incubated for an additional 20 hours at 37°C. After incubation, cells were collected and subjected to analyses.

### 2.4. mRNA Quantification Procedures

Total RNA was isolated from cultured cells using an RNeasy Mini Kit (Qiagen) according to the manufacturer's procedure. Gene expression was evaluated by a two-step quantitative reverse transcription-PCR method. Reverse transcription of RNA samples was performed by using a High Capacity cDNA Reverse-Transcription Kit (Applied Biosystems, Foster City, CA), and quantitative PCR was performed using a Fast TaqMan Gene Expression Assay (Applied Biosystems). The mRNA levels of target genes were normalized to the GAPDH mRNA level. The following primer sets were used for quantitative PCR. For human apoB and GAPDH, assay IDs of Hs01071209_m1 and Hs02758991_g1 were used, respectively.

### 2.5. Western Blotting

Two days after transfection, the cultures were subjected to centrifugation at 4°C, 10,000 rpm for 15 min. Each supernatant was collected into an Amicon Ultra-4 Centrifugal Filter Ultracel PL-10k (Millipore) and centrifuged at 4°C at 3,000 rpm for 1 h, and then each supernatant was added to individual Vivaspin 500 units (Sartorius Stedim Biotech) and centrifuged at 4°C at 3,000 rpm for 0.5 h. Each sample (9 *μ*L) was added to 9 *μ*L of Novex Tris-Glycine SDS Sample Buffer (2x) (Invitrogen) and applied to a 3–8% NuPAGE Tris-Acetate Gel (Invitrogen). Electrophoresis was performed at 180 V for 130 min. The separated proteins were transferred to a PVDF membrane (Millipore) at 220 mA for 120 min. Membranes were then incubated with 10 mL of blocking buffer (Blocking One; Nacalai Tesque) for 12 h at 4°C. Membranes were successively incubated with primary antibody of anti-human ApoB antibody (R&D Systems) for 80 min at room temperature. Then, each membrane was washed with PBS containing 0.1% tween (PBST) 4 times. Membranes were incubated with goat anti-mouse IgG-HRP antibody (Santa Cruz Biotechnology) for 80 min at room temperature. Chemiluminescent detection was performed using an ECL Advance Western Blot Detection Kit (Amersham Biosciences) according to the manufacture's procedure. Bands were visualized using an LAS-4000 mini (Fujifilm).

### 2.6. Statistics

 Application of linear regression techniques to plots of the expression of apoB mRNA levels versus the logarithm of transfection concentration allows estimation of the coefficient (slope) and intercept values using the following equation: apoB mRNA = Coefficient∗log (Concentration) + Intercept. These estimates can be useful guides for the efficacies of the AONs. Linear regression analysis was applied to 500 bootstrap sample sets obtained from three independent cellular assays. The coefficient and intercept of each regression line were compared using Tukey's test or one-sample *t*-test.

## 3. Results and Discussion

To better understand the effect of strand shortening on 2′,4′-BNA^NC^-based AONs, a series of 2′,4′-BNA^NC^-based antisense oligonucleotides of 10- to 20-nt-long phosphorothioate gapmers and 2′,4′-BNA/LNA-counterparts were synthesized (Table 1). Formation of a stable duplex with the target mRNA is a minimum essential step of the onset of an antisense effect. We first evaluated the thermal stability of the duplex formed between each modified AON and the single-stranded 20-nt-long oligoribonucleotide complementary to ApoB-LNA-20 and ApoB-NC-20. As expected, the longer the strand or the greater the number of modifications, the higher the *T*_*m*_ value. Moreover, the *T*_*m*_ values of 2′,4′-BNA^NC^-based AONs surpassed those of their 2′,4′-BNA/LNA-based counterparts for any given length (Table 1), in good agreement with previous reports [11, 17]. Note that the exact *T*_*m*_ values of LNAs in Table 1 are different from those given by Straarup et al., due to differences in composition of the measurement buffer solutions and in the length of the complementary RNAs between the two studies.

**Figure 2:**
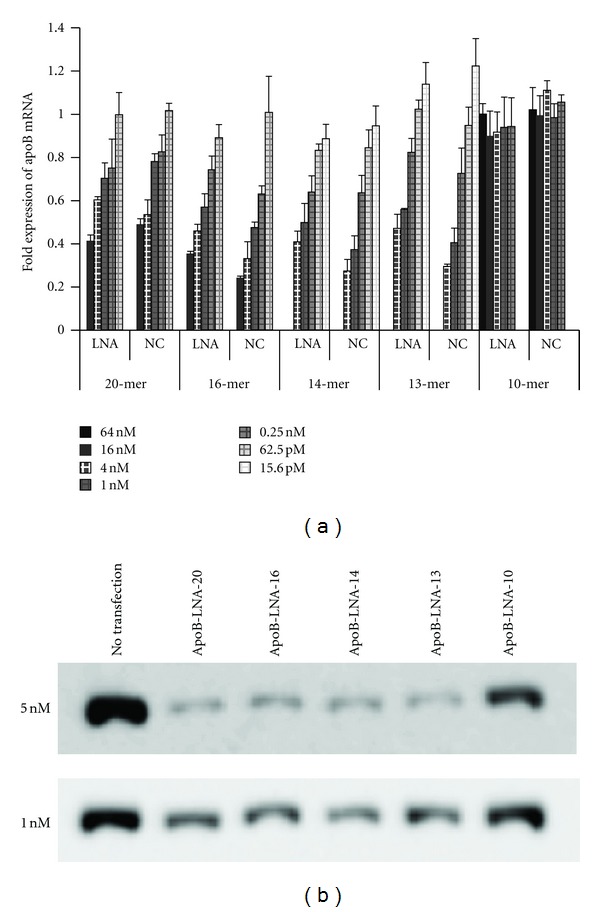
*In vitro *silencing properties of BNA-based AONs. (a) Various concentrations (15.6 pM–64 nM) of AONs were introduced into Huh-7 cells using Lipofectamine 2000. After 24-h incubation, the cells were collected, and the expression levels of apoB mRNA were determined. Data represent means ± SD. (b) Reduction of Apo B protein levels in the culture medium following transfection was confirmed by western blotting.

**Figure 3:**
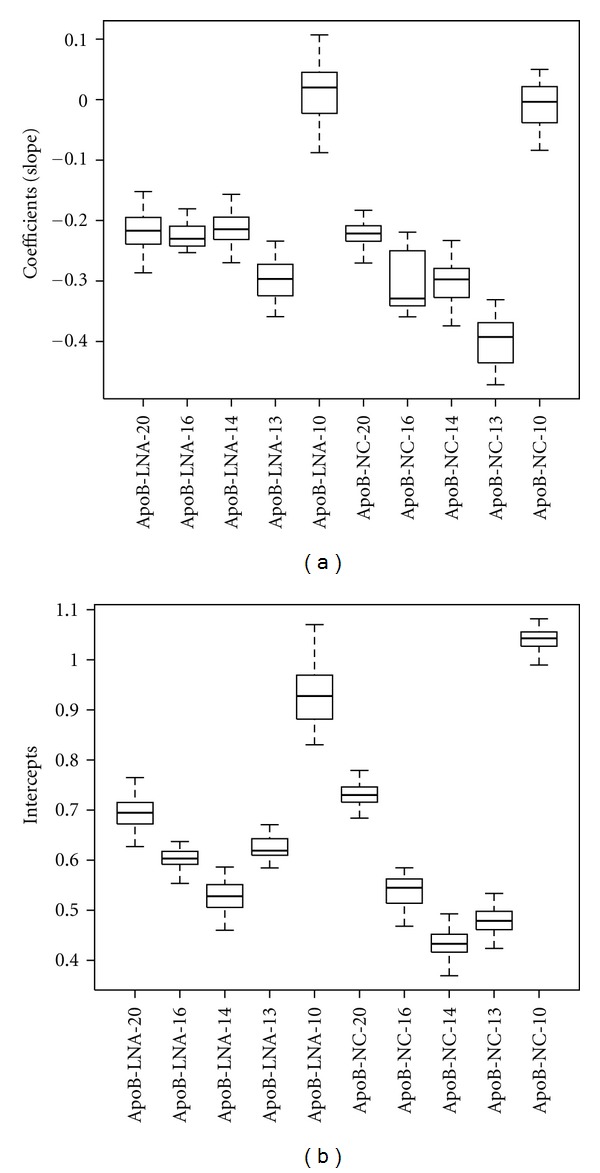
Boxplots of coefficients and intercepts of regression lines obtained from cellular assay data. Bold lines in the box indicate medians. Top and bottom lines of the box indicate upper and lower quartile points, respectively. Top and bottom lines in contact with a dotted line indicate the highest and lowest data point, respectively. Open circles indicate outliers.

 We next used *in vitro* mRNA silencing assays to estimate the potency of 2′,4′-BNA^NC^-based AONs and to compare their potency directly to the corresponding 2′,4′-BNA/LNA-based AONs. We used the Huh-7 human hepatoma cell line, which expresses high levels of apoB mRNA in cells and secrets its protein into the medium. Each AON was introduced using standard lipofection procedures. All the AONs, except the 10-mers, ApoB-LNA-10, and ApoB-NC-10, reduced apoB mRNA and protein expression (and hence secreted protein) levels in a dose-dependent manner in the cells and culture medium, respectively, (Figures 2(a) and 2(b)). ApoB-LNA-10 did not reduce apoB mRNA levels even at concentrations above 64 nM. This may be because ApoB-LNA-10 did not bind target mRNA at 37°C due to lack of affinity. ApoB-NC-10 also did not reduce apoB mRNA expression, despite its higher *T*_*m*_ value compared to that of ApoB-LNA-10. Application of linear regression techniques to plots of the expression of apoB mRNA levels versus the logarithm of transfection concentration allowed estimation of the coefficient (slope) and intercept values. Statistical comparison of these parameters between arms with the identical length would provide useful guides for the efficacies of the AONs. One-sample *t*-tests revealed that the coefficients (slopes) of the 10-mer AONs had statistically insignificant downward slopes, suggesting that these 10-mers have little or no silencing effect (Figure 3).

A length-dependent decrease in potency in 14- to 20-nt-long 2′,4′-BNA/LNA and 2′,4′-BNA^NC^-based AONs was observed (Table 1 and Figure 1). In each series of AONs, the ApoB-LNA-14 and ApoB-NC-14 were the most potent, whereas the ApoB-LNA-13 was the most potent AON reported by Straarup et al. [20]. IC_50_ values of the 15-, 14-, 13-, and 12-mer AONs were so close to each other (*≈*0.5 nM) that Straarup et al. confirmed the order by conducting an *in vivo* silencing study and showed that 12- and 13-mer AONs are the most potent. In contrast, we confirmed the order of *in vitro* silencing effects of all the entries in Table 1 by using larger 12-well culture dishes. These differences in experimental conditions (*in vivo* versus *in vitro*) may explain the differences in the IC_50_ values of 2′,4′-BNA/LNA-based AONs and their order of potency. An *in vivo* study might be necessary to estimate the true order. Nevertheless, we observed in both chemistries that shorter AONs (16-, 14-, and 13-mer) are statistically significantly more potent than 20-mer AONs. Because the 10-mers did not show any activity, activity is positively correlated with binding affinity and indicates the presence of a “threshold affinity.” However, longer AONs with higher affinities did not exhibit higher activities. This suggests the presence of a “length penalty” [20, 22] and the presence of an “optimal affinity,” which might be a more appropriate description than threshold affinity. The variables that independently govern “length penalty” and “optimal affinity” remain largely unknown. However, plasma and intracellular proteins preferentially bind to the phosphorothioate internucleotide linkages, and these linkages are known to control RNase H activity [23–26]. Thus, the number of phosphorothioate linkages is partly related to the “length penalty.”

**Figure 4:**
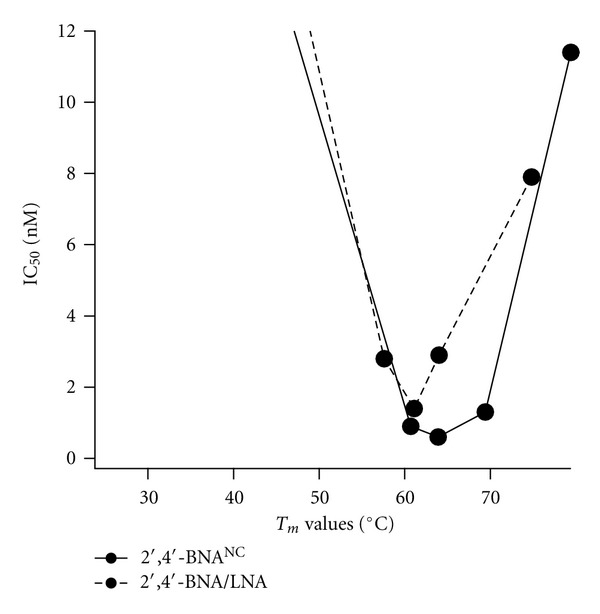
Relationship between target affinity and antisense potency. IC_50_ as a function of *T*_*m*_ values of 2′,4′-BNA^NC^-based AONs (solid line) and 2′,4′-BNA/LNA-based AONs (dotted line).

Surprisingly, when compared to the identical length 2′,4′-BNA/LNA-based counterpart, the corresponding 2′,4′-BNA^NC^-based AON showed stronger inhibitory activity. The differential in inhibitory activity is more pronounced in shorter (13-, 14-, and 16-mers) AONs than in the 20-mer (Table 1 and Figure 4). Indeed, as shown in Figure 3, statistical comparison using the Tukey test of the coefficients and intercepts of the regression lines, which indicate how efficiently and strongly AONs reduce apoB mRNA, revealed that 2′,4′-BNA^NC^-based 13-, 14-, and 16-mers exhibit significantly stronger inhibitory activity than their 2′,4′-BNA/LNA counterparts. These potency differences could not be explained simply by the higher *T*_*m*_ of the 2′,4′-BNA^NC^-based AONs compared to their 2′,4′-BNA/LNA-based counterpart. 2′,4′-BNA^NC^-based AONs might exhibit less “length penalty” than their 2′,4′-BNA/LNA-based counterpart. A weaker affinity for protein binding or lower RNase H inhibitory activity of 2′,4′-BNA^NC^-based AONs could cause this potency difference. On the other hand, Figure 4 shows a plot of IC_50_ versus *T*_*m*_, in which ApoB-NC-20, which has the highest affinity, exhibited worse IC_50 _value than ApoB-LNA-20. These findings suggest that very high affinity possibly undermines antisense potency, although binding affinity to the target generally correlates positively with potency in the case of traditional small molecule drugs. The precise onset mechanism giving rise to this phenomenon remains unclear due to the lack of experimental data. However, the U- or V-shaped curves in Figure 4 clearly indicate that the potency of AONs is the result of a delicate balance of reward term and penalty term. Thus, fine adjustment to the optimal affinity and elimination of the penalties such as excess protein binding and RNase H inhibitory activity could result in superior efficacy, represented as the lowest point on the U- or V-shaped curve.

 In conclusion, we have shown that 2′,4′-BNA^NC^-based AONs targeting apoB mRNA have higher binding affinities to the target RNA than do 2′,4′-BNA/LNA-based AONs. Additionally, *in vitro* transfection studies revealed the superior silencing effect of short 2′,4′-BNA^NC^-based AONs (<20-nt-long), indicating that 2′,4′-BNA^NC^ may have advantageous properties as short antisense drugs. We are currently investigating the potential and safety of 2′,4′-BNA^NC^-based AONs as therapeutic drugs.

## Figures and Tables

**Table 1:** Oligonucleotides used in this study.

ID	Sequence	*T*_*m*_ (°C)	IC_50_ (nM)
ApoB-LNA-20	5′-TTCAGcattggtattCAGTG-3′	75 ± 0.8	7.9 ± 1.7
ApoB-LNA-16	5′-CAGcattggtatTCAG-3′	64 ± 0.8	2.9 ± 0.4^a^
ApoB-LNA-14	5′-AGCattggtatTCA-3′	61 ± 0.2	1.4 ± 0.5^b^
ApoB-LNA-13	5′-GCattggtatTCA-3′	58 ± 0.2	2.8 ± 0.8^a^
ApoB-LNA-10	5′-CattggtatT-3′	25 ± 1.4	N.D.
ApoB-NC-20	5′-*TTCAG*cattggtatt*CAGTG*-3′	79 ± 1.0	11.4 ± 3.0
ApoB-NC-16	5′-*CAG*cattggtat*TCAG*-3′	69 ± 0.8	1.3 ± 0.3^b^
ApoB-NC-14	5′-*AGC*attggtat*TCA*-3′	64 ± 0.3	0.6 ± 0.1
ApoB-NC-13	5′-*GC*attggtat*TCA*-3′	61 ± 0.1	0.9 ± 0.1
ApoB-NC-10	5′-*C*attggtat*T*-3′	32 ± 0.4	N.D.

2′,4′-BNA/LNA was shown in uppercase and 2′,4′-BNA^NC^  was in italic. Natural DNA was shown in lowercase. All the linkages are phosphorothioated. We measured *T*_*m*_ and IC_50_ values of all entries. *T*_*m*_ values were determined in three independent experiments (±SD). Nondetectable IC_50_ values, due to low potency, were marked ND. ^a,b^Pairs of two IC_50_ values with superscript letters are NOT statistically significant.
